# Cooperativity of co-factor NR2F2 with Pioneer Factors GATA3, FOXA1 in promoting ERα function

**DOI:** 10.7150/thno.34874

**Published:** 2019-08-21

**Authors:** Guojuan Jiang, Xinrui Wang, Dandan Sheng, Lei Zhou, Yang Liu, Congling Xu, Suling Liu, Ji Zhang

**Affiliations:** 1 State Key Laboratory of Medical Genomics, Rui Jin Hospital, Shanghai Jiao Tong University School of Medicine, Shanghai 200025, P.R.China; 2 Key Laboratory of Breast Cancer in Shanghai, Cancer Institute, Department of Breast Surgery; Institutes of Biomedical Sciences; Innovation Center for Cell Signaling Network; Fudan University Shanghai Cancer Center, Shanghai 200032, P.R.China

**Keywords:** Breast cancer, ERα, NR2F2, FOXA1, GATA3

## Abstract

Estrogen receptor α (ERα) drives growth in the majority of human breast cancers by binding to regulatory elements and inducing transcriptional events that promote tumor growth. ERα binding activity largely depends on access to binding sites on chromatin, which is facilitated in part by Pioneer Factors (PFs). Transcription factors operate in complexes through thousands of genomic binding sites in a combinatorial fashion to control the expression of genes. However, the extent of crosstalk and cooperation between ERα pioneer factors and more collaborative transcription factors in breast cancer still remains to be elucidated systematically.

**Methods**: Here, we determined the genomic binding information of 40 transcription-related factors and histone modifications with ChIP-seq in ENCODE and integrated it with other genomic information (RNA-seq, ATAC-seq, Gene microarray, 450k methylation chip, GRO-seq), forming a multi-dimension network to illuminate ERα associated transcription.

**Results**: We show that transcription factor, NR2F2 binds to most sites independently of estrogen. Perturbation of NR2F2 expression decreases ERα DNA binding, chromatin openning, and estrogen-dependent cell growth. In the genome-wide analysis, we show that most binding events of NR2F2 and known pioneer factors FOXA1, GATA3 occur together, covering 85% of the ERα binding sites. Regions bound by all the three TFs appeared to be the most active, to have the strongest ERα binding and to be enriched for the super enhancers.

**Conclusions**: The ERα binds to pre-accessible sites containing ERE elements bound by the three transcription factors (NR2F2, FOXA1 and GATA3).The three genes were also identified to correlate with decreased metastatic potential in patient cohorts and co-regulate each other. Together, our results suggest that NR2F2 is a cofactor with FOXA1 and GATA3 in ERα-mediated transcription.

## Introduction

It's becoming increasingly clear that transcription is mediated by a limited cohort of transcription factors with combinatorial fashion to form transcription complex and organize the large diversity of gene-expression patterns to the linage commitment cell types 1-4. Nuclear receptors, including the glucocorticoid, androgen, and estrogen receptors, interact with a large repertoire of transcription factors 1, 5-8. The consequences of these interactions have largely been interpreted in the context of changes in interactions with the general transcription apparatus 9, 10. Estrogen receptor α (ERα) in breast cancer is the same to this by interacting with numerous associated proteins. ERα is a key transcription factor determining the ER positive cell identity and drives growth in over 70% of ER positive breast cancers. Differences in enhancer occupancy by ERα contribute to the diverse expression profiles and clinical outcome observed in breast cancer patients 4, 11. However, ERα binding to DNA is mediated, in most cases, via the estrogen responsive element (ERE) DNA motif, but accessibility to this motif requires the pioneer factors to open the chromatin and facilitate ER-chromatin interactions. Pioneer factors (PFs) have been described as a class of proteins that can bind to nucleosomal DNA to create accessible binding sites for additional transcription factors during development 12. Several protein families have been shown to possess these properties, including forkhead box A, GATA, and TLE. FoxA1, with the winged-helix domain that it shares with the H1 linker histone, has been published as a pioneer factor of ER to interact with compact chromatin, modulating chromatin structure as an early event 13-15. The GATA family proteins, which possess two zinc-finger domains, play critical roles across development and differentiation, contributing to lineage commitment. Importantly the DNA-binding domains of GATA family proteins are structurally distinct from FOXA1, implying that mechanism(s) to create open chromatin may differ. The individual role of pioneer factor in determining selective binding patterns of ERα may also different due to the different binding structures and motifs 16.

This transcription factor colocalization and complexes at enhancer elements govern the genomic circuitry, and these can greatly influence on chromatin accessibility and transcription programs 17-20. Pioneer factors that disrupt the closed nucleosome conformation and initiate silenced genes play important in biological development and cell reprogramming. So, it seems more likely and essential for pioneer factor to work together with different combination to establish cell-specific binding sites for cell identity responses to diverse signaling inputs. Thus, it was hypothesized that it is not a certain pioneer factor alone but several individual pioneer factors working together to optimally direct the ERα binding. Such research has been well characterized in embryonic stem cells 21. Nine transcription factors including cell reprogramming pioneer factors (OCT4, SOX2, and KLF4) and other co-factors (Nanog, DAX1, REX1, c-MYC, ZPT281, and NAC1) form transcriptional hierarchy to maintain pluripotency. Promoters bound by few of the nine factors tend to be inactive or repressed; whereas promoters bound by more than four factors are largely active. In breast cancer cells, published data shows Fork head (FOX) and GATA3 DNA motifs are enriched within ERα-binding regions 14, 22. However, the genomic binding interplay between them is not fully explored. Considering the complexity of transcriptional regulation, it is likely there are many more interactions of pioneer factors or other co-factors that are required for ERα function, which currently remains unknown.

We have utilized genome-wide analysis of ChIP-seq data of more than 30 transcription factors/histone modification markers in ER positive breast cancer model cell line MCF-7, from ENCODE (The Encyclopedia of DNA Elements) 23 and our group (see Table S1), to gain global insight into the interactome of ERα. Here, we report a potential novel ERα co-factor, NR2F2. The novel discovered co-factor NR2F2 is one of the two highly homologous subtypes of COUP-TFs: COUP-TFI (EAR-3 and NR2F1) and COUP-TFII (ARP-1 and NR2F2). COUP-TFs, which are chicken ovalbumin upstream promoter transcription factors, are members of the nuclear receptor superfamily 24. They are referred to as orphan receptors because their ligands have not yet been identified. Since the discovery of COUP-TFs in 1986, extensive studies have demonstrated their crucial functions in a variety of developmental processes 24, 25. Recently, increasing evidence has highlighted that COUP-TFs, specifically COUP-TFII, play important roles in the progression of various cancers 24, 26-28. In breast cancer, high NR2F2 expression was reported to be related to increased survival, and knockdown of NR2F2 promoted breast cancer cell migration and invasion *in vivo* 29. IHC staining of NR2F2 in breast cancer primary biopsy tissue with metastatic lymph nodes demonstrated that a reduction in COUP-TFII was associated with an increased tumour stage and worse progression of malignancy in breast cancer. Analysis of seven previously used breast cell microarray datasets (NKI, GSE7390, GSE47561, GSE16446, GSE25066, GSE2034, and GSE6532) and TCGA dataset revealed that the NR2F2 expression level was inversely related to mesenchymal markers and that NR2F2 had a negative impact on the expression levels of TGF-β targeted genes 29. Thus, NR2F2 inhibits the TGF-β-dependent epithelial-mesenchymal transition in breast cancer. In contrast, COUP-TFIIs play a crucial role in inducing indolent prostate cancer to develop into metastatic-prone cancer by directly interacting with SMAD4 to destroy the TGF-β-induced growth barrier 26. In addition to prostate cancer, increasing evidence suggests that COUP-TFII may play an important role in ovarian and colon cancer progression 27, 28. In our study, we showed that NR2F2 and known pioneer factor, FOXA1 and GATA3, bind together across the genome, covering 85% of the ERα binding sites in a genome-wide analysis. NR2F2 together with GATA3 and FOXA1 cooperatively assiciate with estrogen receptor binding by establishing a pre-existent super-enhancer landscape that contained ERE motifs to mediate related biological processes, such as proliferation, metastasis and regulation of genes driving the ER core function. In addition, we found that the expression of GATA3, NR2F2, and FOXA1 strongly correlate with ER positive histological state in patients and cell lines and hypermethylation in promoter regions in ER negative samples contributed to the low expression in these patients. Our findings interpret the mechanism by which the ERα functions through pioneer factor complex with chromatin and may develop novel potential therapeutic targets for hormone-therapy resistant.

## Materials and Methods

### Cell Culture Conditions

The MCF-7, ZR-75-1, T-47D, MDA-MB-453, SKBR3, SUM159, MDA-MB-435, MDA-MB-231 and LM-2 breast cancer cell lines have been utilized in this study. Cell culture conditions is provided in the standard procedures.

### Generation of stable cell line

Lentiviruses were produced by transient transfection of 293T cells with pHAGE, psPAX2, and pMD2.G plasmids. The virus containing medium and polybrene (at a final concentration of 8 μg/mL) were added to MCF-7 cells. Infected cells were selected with 0.5 μg/mL puromycin. Only early passage cells were used in all experiments. Cells expressing either GFP or the tag alone (including the TEV protease recognition site) were used as a control cell line.

### Chromatin Immunoprecipitation

ChIP experiments have been performed as per standard protocols 30, 31. Briefly, cells were treated with either 100 nM E2 or left untreated for 1 hour. The following antibodies have been utilized: ER (Ab-10, sigma), NR2F2 (sc-271940, Santa Cruz Biotechnology) and FOXA1 (ab23738 Abcam), H3K4me1, H3K4me3, H3K27ac.

### ChIP-seq analysis

Data was analyzed and processed by a unified procedure to eliminate the bias by data processing (Detailed information of data shown in Table S1). All ChIP-seq data sets were aligned using Bowtie (version 2.2.4) 32 to build version hg19 of the human genome. Data used in this manuscript can be found in Table S1. We used the MACS version 2.1.1(model-based analysis of ChIP-seq) peak finding algorithm to identify regions of ChIP-seq enrichment over background. The operating parameters are set as follows: macs2 callpeak -t tfile -c cfile -n out-name --outdir out-dir -g hs -f BAM -m 10 40 -p 1e-6. BAMPE is used with paried end reads. We developed a simple script to calculate the normalized read density of ChIP-seq data in any given region. Each given site was extended to 2kb from the center of the vertex and the 4kb region was divided into 40 bins in units of 100bp. The density of reads per bin(100bp) was calculated. In order to be able to compare between multiple groups, the density of reads was normalized to the total mapped reads producing signal in units of reads per 10 million mapped reads per 100 base pair(10*rpm/bp). Enhancers were defined as regions of ChIP-seq enrichment for H3K27ac. Super enhancer was defined and calculated as described in 33, 34.

### RNA-Seq sample preparation and data analysis

RNA-seq was performed as described previously with slight modification 35, 36. The MCF-7 cells were grown in the phenol red-free medium supplement with 10% charcoal/dextran-treated fetal bovine serum for 3 days, followed by treatment of vehicle or 10 nM estrogen for 12 hours. Total RNA was prepared from approximately 30,000-50,000 cells by using TRIzol following the manufacturer's protocol (Life Technologies). Total RNA was subsequently processed to generate an mRNA-seq library using a TruSeq SR mRNA sample prep kit (FC-122-1001; Illumina). The libraries were sequenced for 50 cycles (single read) with a HiSeq 2000 or HiSeq. Stable MCF7 cells were transfected with shRNA and cultured for 72 hours in phenol red-free RPMI 1640 supplemented with charcoal-stripped FBS and 1% penicillin-streptomycin (steroid-deprived medium). E2 (Sigma, no. E8875-250MG) dissolved in ethanol (vehicle) were used at a final concentration of 100 nM for all experiments. Cells were subsequently treated with 100nM estrogen (E2) or control (Veh) for 6 hrs.

Sequence reads were mapped onto hg19 genome using TopHat2.1.0 35, 37. Gene expression values (FPKM; fragments per kilobase exon per million mapped reads) were calculated with Cufflinks 2.2.1 (Trapnell et al., 2012). BigWig tracks were generated from Bam files and converted into bedGraph format using bedtools 38. These were further reformatted with the IGV tool. The differential gene expression was determined by DEseq using 2-fold change, with p value <0.05 as threshold (Anders and Huber, 2010). Downstream analyses and heatmaps were performed with R 3.0.1 (R Core Team, 2014) and custom R program.

### ATAC-Seq preparation and data analysis

ATAC-seq was performed according to a published protocol 39 with minor modification. Fifty thousand cells were pelleted and washed with 50 ml 13 PBS, followed by treatment with 50 ml lysis buffer (10 mM Tris-HCl [pH 7.4], 10 mM NaCl, 3 mM MgCl2, 0.1% IGEPALCA-630). After pelleting the nuclei by centrifuging at 500 3 g for 10 min, the pellets were re-suspended in a 40-ml transposition reaction with 2 ml Tn5 transposase (FC-121-1030; Illumina) to tag and fragmentalize accessible chromatin. The reaction was incubated at 37℃ with shaking at 300 rpm for 30 min. The fragmentalized DNAs were then purified using a QIAGEN MinElute kit and amplified with 10 or 11 cycles of PCR based on the amplification curve. Once the libraries were purified using a QIAGEN PCR cleanup kit, they were further sequenced for 50 cycles (paired-end reads) on a HiSeq 2500.

ATAC-seq reads for each sample were mapped to hg19 genome using Bowtie 2.2.4 32. In all cases, redundant reads were removed using FastUniq 40, and customized Python scripts were used to calculate the fragment length of each pair of uniquely mapped paired-end (PE) reads. The fragment sizes distribute similar to previously published data. Only one mapped read to each unique region of the genome that was less than 175 bp was kept and used in peak calling. Regions of open chromatin were identified by MACS (version 1.4.2) 41 using a p-value threshold of 1 3 10_5. Only regions called in both replicates were used in downstream analysis. Peak intensities (''tags'' column) were normalized as tags per 10 million reads (RP10M) in the original library. Downstream analysis and heatmap generation were performed with the Hypergeometric Optimization of Motif EnRichment program (HOMER) version 4.8 3 and R 3.4.0 42.

## Results

**Figure 1:**
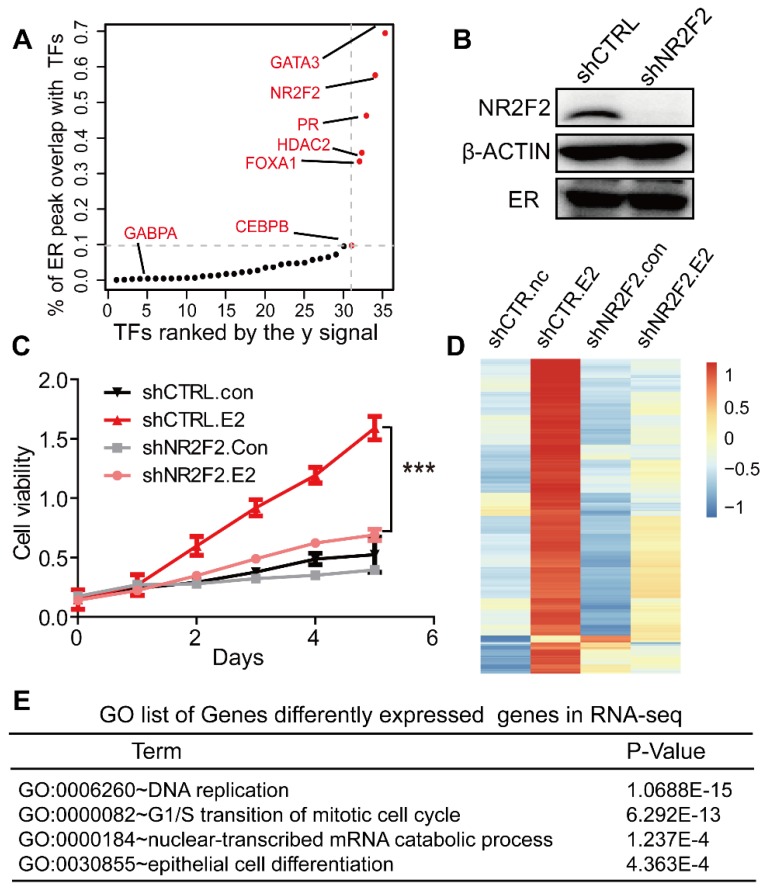
** NR2F2 is essential to the oestrogen response in cell line MCF7. (A)** Distribution of the overlap (value of y) of all transcription factors with the ERα. The y value is generated using the overlap peak number of the ERα and the corresponding factor, divided by the total numbers of merged peaks of each transcription factor and the ER. **(B)** NR2F2 stable depletion via lentivirus-delivered shRNA effectively reduces its protein levels but does not alter ERα expression. **(C)** MCF-7 breast cancer cells depleted of NR2F2 failed to proliferate in response to oestrogen stimulation compared to cells subjected to the control treatment to a certain degree. **(D)** RNA-seq was performed on MCF-7 cells transfected with the control or NR2F2 shRNA and stimulated with or without E2 for 12 h. **(E)** Gene ontology (GO) functional categories for differently expressed genes by RNA-seq. Genes encoding factors important for DNA replication, the G1/S transition, and epithelium were enriched.

To explore the landscape of ERα transcription-associated factors, we overlapped ER binding sites with other transcription factors and used the overlapped peak numbers divided by all ER peak counts to calculate the overlap coefficient to assess the overlap between ERα and each factor. We followed the super-enhancer algorithm to identify the first few transcription factors with the highest overlap with ERα 33, 34. As expected, our screen identified binding sites for previously reported collaborative factors of ERα, such as FOXA1, GATA3 (Figure 1A and Table S3) 14. In addition, transcription factor NR2F2 (nuclear receptor subfamily 2 group F member 2) was also identified. Our data reveals that 40% of all ER-binding events overlap with FOXA1‑binding events, 57% overlap with NR2F2 binding events, 69% overlap with GATA3 binding events in MCF-7 (Figure 1A). Previous reports show that FOXA1 behaves as pioneer factor for ER and AR binding genome-wide both in breast and prostate, and GATA2 is also required for AR binding in prostate^12^, ^43^, ^44^. To further confirm the accuracy of the relevant transcription factors we identified, we screened the interactome of AR and PR in the same way. Finally, FOXA1, GATA3, and NR2F2 also overlap with the AR, PR with highest degree (Figure S1A, S1B and Table S4, Table S5). Our research focused on the common three transcription factors. Previous studies showed that high NR2F2 transcription level was correlated with favourable overall survival. Little is known about the interaction between NR2F2 and ERα 45. The mechanism by which they interact need more studies.

### NR2F2 is essential to the oestrogen response in MCF-7 cells

To address the functional relationship between NR2F2 and ERα, we assessed the role of NR2F2 in oestrogen-induced growth in ER positive cell line MCF-7. NR2F2 protein level was significantly depleted in MCF-7 transfected with shRNAs (Figure 1B). The MTT experiment showed that inhibition of NR2F2 prevented the oestrogen-induced proliferation of MCF-7 cells (Figure 1C). We expanded our knockdown studies by performing RNA-seq for MCF-7 cells transfected with control or NR2F2 shRNAs with or without E2 with two bilogical replicates. Interestingly, depletion of NR2F2 affected the expression of a significant number of E2-induced genes (Figure 1D). As shown in Figure 1D, these oestrogen upregulated genes were no longer activated due to NR2F2 knockdown. Gene ontology analysis revealed that the differentially expressed genes are linked to biological processes related to DNA replication, cell cycle and cell differentiation (Figure 1E). Furthermore, to measure the effects of NR2F2 knockdown on proliferation of other breast cancer cells lines, we chose another two ER positive cell lines BT-474 and T-47D to perform MTT assay. The results show that knockdown of NR2F2 also reduces the estrogen induced growth in BT-474 and T-47D (Figure S1C to S1E). Overall, these results demonstrate that NR2F2 is required for efficient transcription of ERα target genes and support a functional role for NR2F2 in mediating oestrogen-dependent cell growth in ER positive breast cancer cell lines.

### Depletion of NR2F2 redistributes ERα binding and reduces accessibility of chromatin

**Figure 2:**
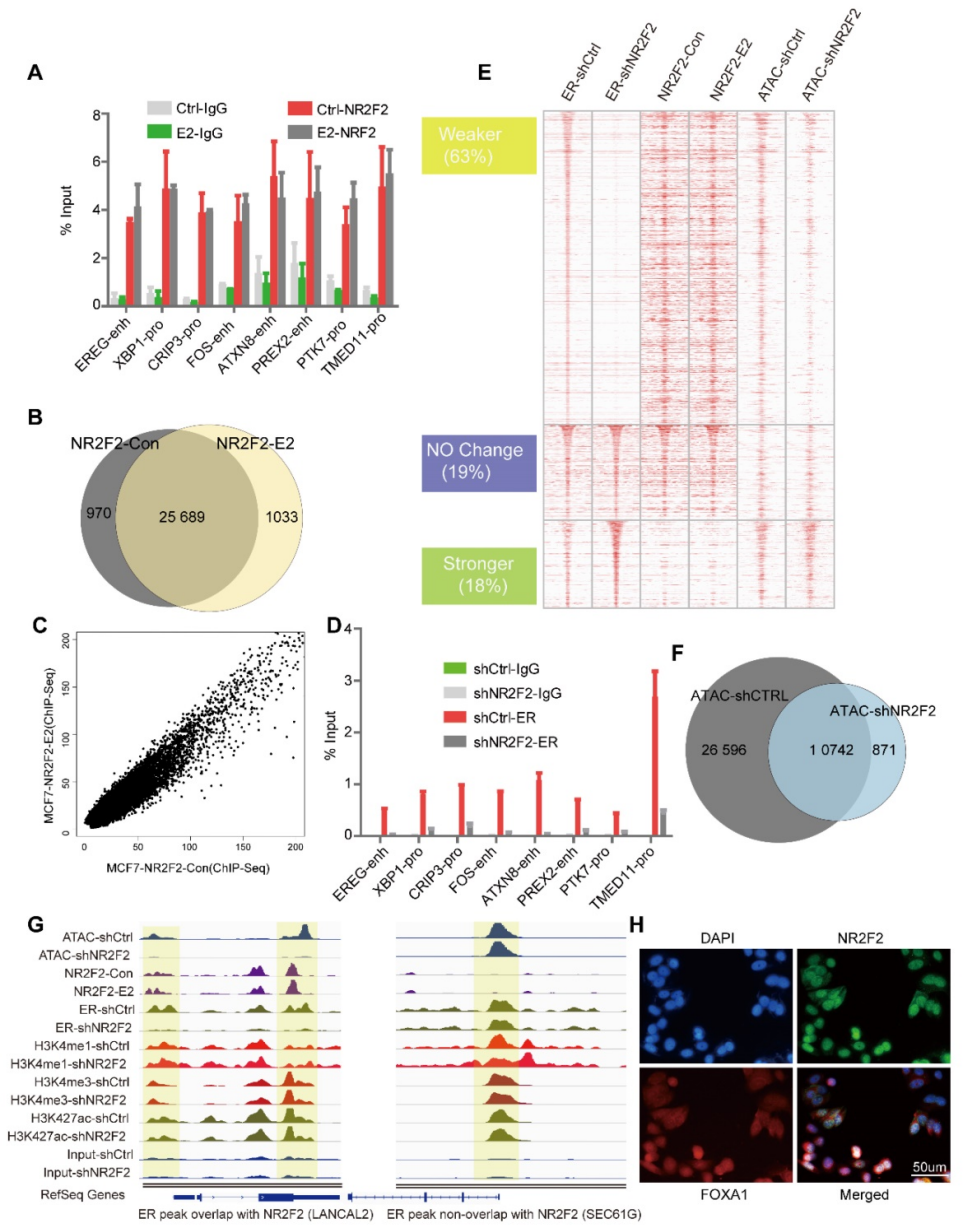
** NR2F2 acts as a co-factor of ERα in MCF7. (A)** The ChIP-qPCR against NR2F2 was performed in MCF-7 cells at given ERBSs treated with or without E2 for 6 hours. The ERα binding signal at the given sites was obviously reduced after NR2F2 knockdown. **(B)** Comparison of NR2F2 binding sites overlap under vehicle or E2 conditions with Venn diagram. **(C)** Scatter plots representing the correlation of peak intensities of NR2F2 before and after E2 stimulation. **(D)** ChIP-qPCR for given ERBSs using an ER antibody was performed in MCF-7 cells transfected with the control or NR2F2 shRNA and treated with E2 for 6 hours. All results represent the average of three independent experiments. **(E)** Heatmap analyses of ChIP-seq signals of NR2F2, ERα, and ATAC-seq in the control and shNR2F2 MCF-7 cells, ranked by ChIP-seq signals of ER. All ChIP-seq signals are displayed from -2.5 kb to +2.5 kb surrounding the center of each annotated ER peak. **(F)** Venn diagram show ATAC peaks across genome with shCtrl and shNR2F2 conditions. **(G)** Representative examples of normalized ATAC-seq signal, ChIP-seq signal of ER and NR2F2, and histone modification profiles in MCF-7 cells at two select ER binding location overlap or non-overlap with NR2F2. (H) Immunofluorescence analysis in MCF-7 cells cultured in the absence of E2 revealed that NR2F2 was localized in the nucleus of MCF-7 cells and overlapped with the pioneer factor FOXA1.

Since GATA3 and FOXA1 have been shown to bind in MCF-7 in a ligand-independent manner, we investigated the potential role of ERα recruiment on NR2F2 binding 16, 46. We performed ChIP-qPCR against NR2F2 at some published ERα binding sites (ERBS) 47 and ChIP-seq genome wide in MCF-7 cells before and after E2 stimulation. ChIP-qPCR results show NR2F2 occupies the given ERα targets prior to E2 treatment (Figure 2A). ChIP-Seq show that majority of sites (25 689) appears to be present in unstimulated states (Figure 2B) and a strong correlation (R^2^=0.9) (Figure 2C) between the peak intensities of NR2F2BS in both conditions. These results suggested that NR2F2 loaded on the chromatin before treatment and remained constant after the addition of E2.

Since NR2F2 associates with chromatin prior to estrogen treatment and its depletion in MCF-7 cells did not affect ERα expression (Figure 1B), we hypothesize NR2F2 may inhibit estrogen-dependent growth by modulating ERα recruitment. We performed first ChIP-qPCR against ERα at the same given ERBS and ChIP-Seq genome wide before and after NR2F2 depletion. ChIP-qPCR results show that NR2F2 silencing (shNR2F2) abrogates ERα recruitment on the given sites compared to control (shCtrl) (Figure 2D). Through analysis, we identified a total of 10410 ERα-binding events, NR2F2 silencing induced a redistributed ERα-binding profile at approximately 80% percentage of all bindings. Results show that 63% of all ERα-binding events had decreased binding affinity, 19% did not change, and 18% of ERα-binding events increased inbinding intensity (Figure 2E). Importantly, ERα sites with decreased binding affinity (Weaker) when NR2F2 is silenced tend to be cobound with NR2F2 in wild-type cells. In contrast, sites that gain ERα-binding affinity (Stronger) in shNR2F2 conditions have limited NR2F2 binding in non-silenced MCF7 cells. This is shown by the averaged binding signal of NR2F2 in the distinct ERα categories (Figure 2E).

Since FOXA1 and GATA3 can remodel chromatin accessibility, we further explored what effect of NR2F2 would have on chromatin properties. ATAC-seq (Assay for Transposase Accessible Chromatin with high-throughput sequencing) is widely used to map chromatin accessibility genome-wide 39, 48. Thus, We perform ATAC-seq without oestrogen stimulation before and after NR2F2 depletion. Results show that ATAC peaks lost 69% after NR2F2 depletion (Figure 2F). At ERα weaker sites, we found a obvious decrease in ATAC-seq signal, which suggest NR2F2 help maintain open chromatin at its binding sites (Figure 2E).

Covalent modifications are a main chromatin property 17. Recent evidence suggests that FOXA1 may favour H3K4me1 deposition via recruitment of the methyltransferase MLL3 49. To test whether NR2F2 favoured histone modification deposition on chromatin, we profiled ChIP-Seq of H3K4me1, H3K4me3, and H3K27ac following NR2F2 depletion in oestrogen-starved MCF-7 cells to gain comprehensive histone medication landscape. However, NR2F2 depletion did not affect deposition of H3K4me1, H3K4me3 and H3K27ac on chromatin in ERα binding sites (Figure S2A). This reveals that NR2F2 alone cannot modulate histone modification. Taken together, our results show that NR2F2 associates with chromatin prior to ERα recruitment and is required for keeping accessible chromatin for most of ERα binding events.

**Figure 3:**
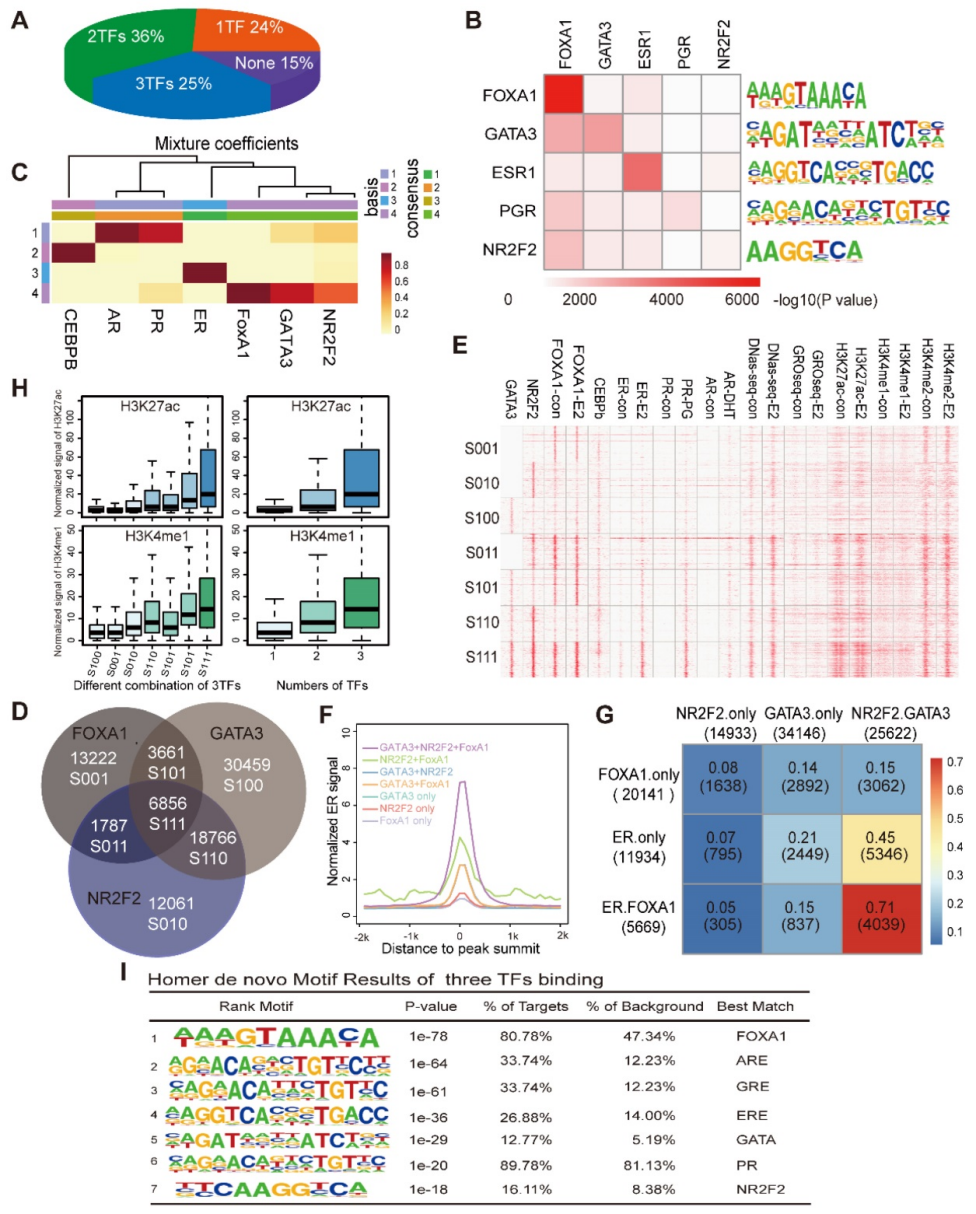
** NR2F2, GATA3 and FOXA1 associate with open chromatin that contains ER motifs. (A)** The proportion of ERα binding sites co-localized by the numbers of PFs (NR2F2, GATA3, and FOXA1) from one to three. **(B)** Motif enrichment of each transcription factor binding site. Rows show the -log10(p-value) of the motif enrichment level of the TFs labelled in the corresponding column using the TF binding sites in the row scanned with Homer. **(C)** The heatmaps visualize the NMF coefficient matrix for the binding signals of the top few transcription factors. The columns show the clustered complexes, and the rows show the coefficient weights of the corresponding TFs for each complex. **(D)** Venn diagram demonstrating the overlap of the ChIP-seq FOXA1 binding patterns with the NR2F2 and GATA3 binding patterns. Each group identified has been notated with the corresponding label and the number of overlapping binding sites. **(E)** Heatmap representing the binding intensity of the corresponding factors in the column, with the specific binding groups characterized by the Venn diagram and labelled with lowercase letters. The heatmap is presented as the number of reads per kilobase per million mapped reads across the position of the reads in a 2 kb region flanking the centre of the peak. **(F)** The distribution of ER signals in the different combinations over a 2 kb region at the correspoi. **(G)** Percentage of merged sites between labelled row factors and column factors. **(H)** The normalized H3K27ac and H3K4me1 signals at the binding patterns identified in the Venn diagrams and by different numbers of TFs over a 2 kb region. The data show that the ERα binding portion is abundantly improved among sites bound by all three factors. **(I)** Motif enrichment of the sites bound by all three TFs with the Homer software.

Immunofluorescence assays against NR2F2 in MCF-7 breast cancer cells deprived of oestrogen further demonstrated its localization to the nucleus (Figure 3H). Although NR2F2 and FOXA1 have similar nuclear distributions, confocal immunofluorescence analysis against NR2F2 revealed that it only partially overlapped with FOXA1.

### NR2F2, FOXA1 and GATA3 associate with stronger ERα binding by cooperatively pre-loading on more accessible chromatin containing ERα motifs

We separately calculated the overlap degree of ERα with NR2F2, GATA3, FOXA1. However, what percentage of ER binding events are synergistically covered by NR2F2, GATA3, FOXA1 remains to be investigated. Thus, we calculated to what degree specific ER-binding regions used one, many or none of the GATA3, NR2F2, and FOXA1. The pie chart showed that 25% of all ER-binding events were bound by all three factors, 36% were bound by two of the the three factors and 24% were bound by one of the three (Figure 3A). Overall, the three transcription factors covered 85% of the ERα binding sites, greatly increase the percentage observed only among FOXA1 binding. Thus, a large degree of cooperativity likely exists between individual pioneer transcription factor.

We also investigated the overall co-occupancy and overlap of the three TFs genome-wide through integrating binding information for FOXA1, GATA3 and NR2F2 and calculated sites bound by different combinations of the three PFs. Motif analysis showed that the peaks of each transcription factor harboured a very significant amounts of motifs of the other factors (Figure 3B). The NMF (Figure 3C) results hinted that the three TFs bound across the genome with a high degree of colocalization.

Analysis of the direct overlap between the binding patterns of the three PFs revealed seven different combinations (Figure 3D, denoted by the category label; the transcription factor order is GATA3, NR2F2, and FOXA1. 0 represents the absence of binding and 1 represents the presence. S100 represents sites only bound by GATA3, and S110 represents sites bound by GATA3 and NR2F2.). The individual binding intensities of the transcription factors and hormone receptors (ER, PR, and AR) with the corresponding treatment, histone modification makers and DNase-seq results were compared via heatmap analysis, and the presence or absence of the three binding events in each group were described by the Venn diagram (denoted by category letters; Figure 3E). Among the different combinations, we observed that GATA3+NR2F2+FOXA1 overlap sites had the highest ER binding signal, percentage and chromatin accessibility (Figure 3E, Figure 3G, and Figure 3F). Interestingly, genome-wide profiling of the DNase-seq signal in the different combinations of the three TFs revealed that the more factors that colocalized to a given region, the higher are the levels of chromatin accessibility, DNase-seq signal, ERa recruitment (Figure 3F) and deposition of the histone modifications H3K4me1, H3K4me2, and H3K27ac (Figure 3H). Interestingly, in the three groups with binding of only one TF, the group with only NR2F2 (S010) binding had the highest DNase-seq, H3K4me1 and H3K27ac signals compared with those of the other two groups (S100 and S001). In the three groups bound by two TFs, the group without NR2F2 binding (S101) has the lowest DNase-seq, H3K4me1 and H3K27ac signals when compared with those of the other two groups (S110 and S011). Based on previous reports and our own results, we can conclude that these three factors associate with chromatin more accessible to recruit the ER. Therefore, we hypothesize that these sites should contain an ER responsive element. As expected, motif scanning of the sites bound by the three factors revealed that in addition to their own motifs, the ER and other steroid receptor motifs had a large degree of enrichment (Figure 3I). FOXA1 alone can bind to the specific DNA motif on the nucleosome core and displace the linker histones, leading to de-compaction of chromatin and facilitating the binding of other TFs. GATA3 can function as a pioneer TF in the mesenchymal-epithelial transition reprogramming event and induce nucleosome eviction, alter local histone modifications and remodel local chromatin architecture thought to be previously inaccessible 15. In our analysis, after NR2F2 depletion, ATAC-seq signal were reduced at ER binding sites. Taken together, our data demonstrate that these three factors synergistically bind with more open chromatin, exposing the ERE motif as an early event to promote ER binding.

### ERα utilizes a pre-established active enhancer landscape co-bound by NR2F2, FOXA1, and GATA3 associated with the ERα core function

**Figure 4:**
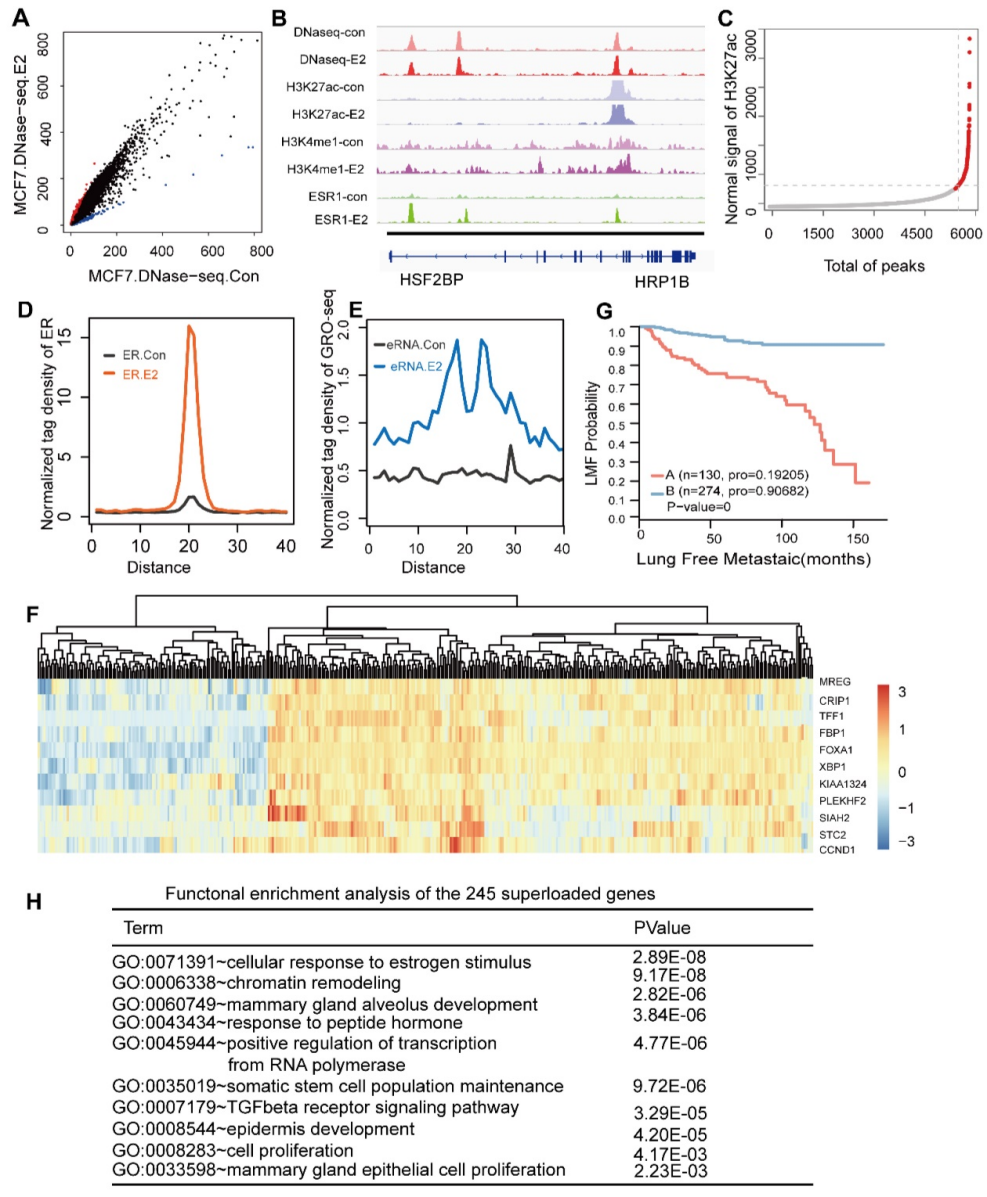
** The ER utilizes a pre-established functional enhancer landscape bound by the PFs associated with the ER core function. (A)** Scatterplot of the DHS peak normalized signal found in either the E2-treated or untreated cells demonstrating new E2-induced sites (red) and sites lost in the untreated cells (blue). **(B)** Shown are examples of DNase I sensitivity, histone modification marker and oestrogen receptor occupancy patterns before and after hormone exposure. **(C)** Distribution of the H3K27ac ChIP-seq signal across the 6856 sites bound by all TFs. H3K27ac occupancy is not evenly distributed at the regions, with a subset of sites (the 279 super-enhancers) containing exceptionally high H3K27ac signals. **(D, E)** Normalized tag density distribution of ER ChIP-seq (D) and GRO-seq (E) signals across the 279 super-enhancer region TFs before and after E2 stimulation. **(F)** The change in the ERα ChIP-seq signal was measured before and after the addition of oestrogen across the 241 aligned genes. The top ten genes with significant differences were used to perform a cluster analysis of the 404 collected patients. The results showed that the patients were obviously clustered into two categories. **(G)** Kaplan-Meier metastasis analysis of these two groups of patients show a significant difference in the prognosis with a p-value of almost zero. (H) Functional enrichment analysis of 241 genes that were uniquely aligned to the genome using the online DAVID software. The results show that these genes are significantly associated with functional modules, such as oestrogen, chromatin remodelling, mammary gland epithelial cell proliferation and mammary acinar growth (p-value<1e-3).

Our analysis suggested that ER binding was not related to nucleosome depletion and the signal heatmap implied that very few alterations in DNase-Seq (Figure 4A) and histone modification (Figure 4B and Figure 3E) occurred after E2 stimulation, in accordance with previously reported analyses 50. Chromatin accessibility remains extremely constant before and after ER binding. Furthermore, GATA3 and FOXA1 have been previously shown to bind in MCF7 breast cancer cells in a ligand-independent manner 14, 16, 46, 51. Our results also show the binding of NR2F2 is almost estrogen independent. Thus, FOXA1/GATA3/NR2F2-chromatin interactions were not influenced by oestrogen treatment and pre-loaded before ERα recruitment to the chromatin.

Previous ChIA-PET studies of the ER have shown that the majority of ER binding sites are distant from the transcription start sites of regulated genes 52. ER occupied distant enhancers and via DNA-looping brings enhancers in spatial proximity of promoters of regulated genes 53. The histone landscape and transcription factor complexes at enhancer elements govern the genomic sites that define cell fate, and these can be greatly influenced by chromatin accessibility formed by pioneer factors 54, 55. As reported, FOXA1 can translate epigenetic signatures into enhancer-driver lineage-specific transcription 8. Additionally, our data showed that most sites co-localized by the ERα and the three transcription factors were enhancer regions. Recent findings indicate that a small portion of enhancer form as super-enhancers, which are associated with key genes that control cell state. Thus, we assessed the super-enhancers in these regions. Among the more than 6000 enhancer regions co-occupied by these three transcription factors, we screened 280 super-enhancers based on the H3K27ac level in these sites (Figure 4C). To determine the functions of the super-enhancers, we calculated the ERα signal before and after E2 stimulation. A dramatic increase in ERα binding was observed at these super-enhancers (Figure 4D). We further investigated whether the effective binding of the ERα to these regions after E2 treatment could active the enhancer. Recent studies have shown that noncoding transcription may be occurring at ERα binding enhancer elements and active enhancers are transcribed to produce non-coding RNAs called enhancer RNAs (eRNAs) 56-58, although these regions can still simultaneously be involved in chromosomal loops 52. Global run-on sequencing (GRO-seq) is the most widely used method to measure nascent RNA and has been applied successfully to study non-coding RNAs, including eRNAs, in recent years. Thus, we integrated GRO-seq using E2 treatment with a consistent dose in published paper 59. The results showed that the GRO-seq signal was substantially increased (Figure 4E), suggesting that upon E2 stimulation the ER was recruited to these super-enhancer on a large scale and contributed to a high transcription level (Figure 4D and Figure 4E).

We further explore the functions of the genes distributed around the scanned super-enhancers. We used proximity to assign the 280 super-enhancers to 241 unique genes (gene list in Table S2). These 241 genes clustered the 404 collected patients into two groups (Figure 4F), which exhibited significant differences in their prognoses (p-value=0) (Figure 4G). GO analysis of these genes implied that they played key roles in the proliferation and development of breast cancer (Figure 4H). The genes were enriched in the TGF-β signal pathway. Previous studies showed that NR2F2 was associated with beneficial clinical outcomes especially metastasis by inhibiting the TGF-β-dependent epithelial-mesenchymal transition 29, which was confirmed in our study.

### NR2F2, FOXA1 and GATA3 are co-expressed in ERα positive breast cancer

**Figure 5:**
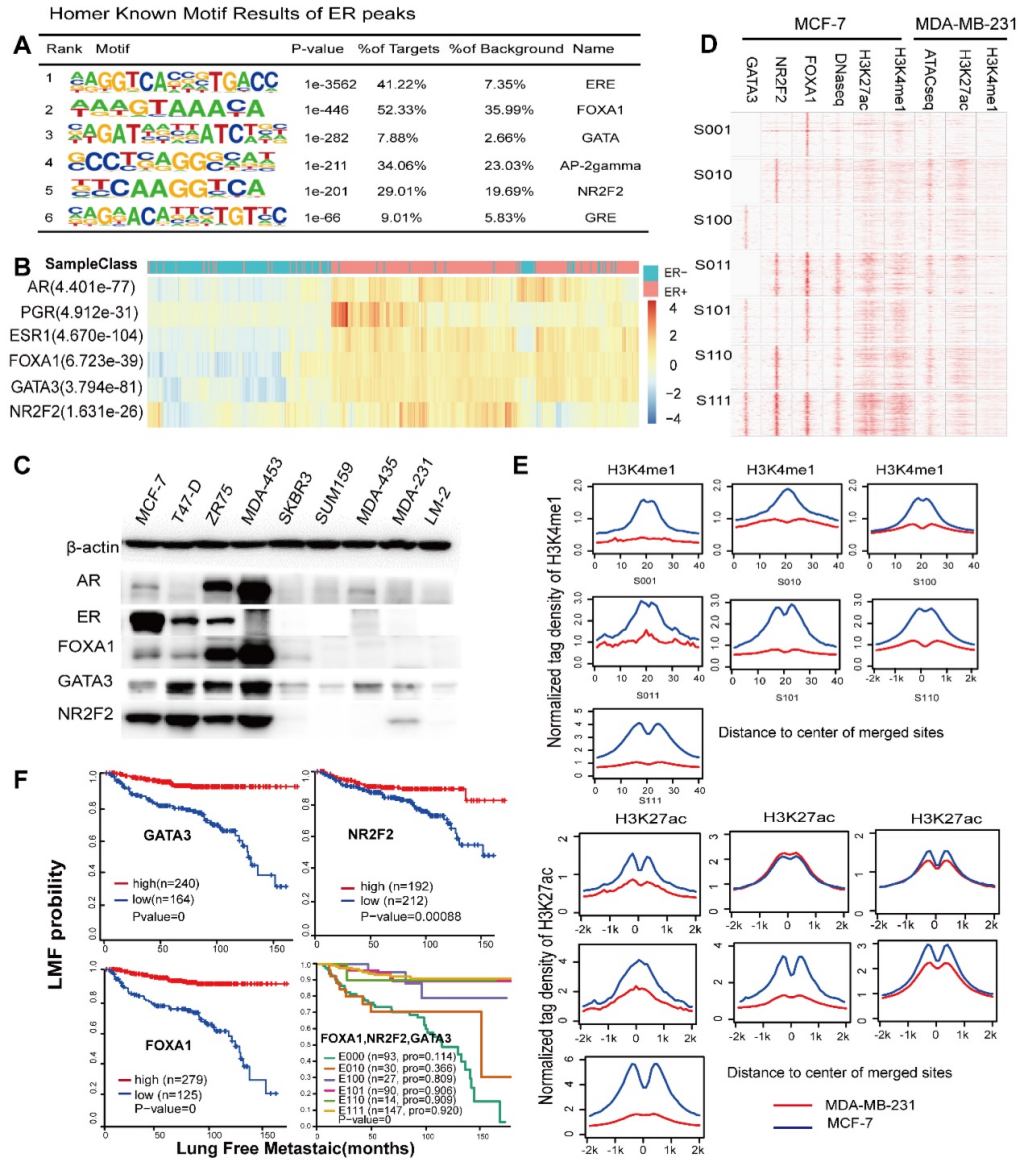
** These factors are required for ESR1+ breast cancer. (A)** Representation of known motif results for ER binding using the Homer software. **(B)** Expression profiles from primary breast tumours reveal that the mRNA levels of the three TFs are correlated with the ERα histological status and mRNA expression. The significant p-value is presented. **(C)** Western blotting experiments of corresponding TFs and hormone receptors were performed on a panel of nine breast cancer cell lines, including ER positive (MCF-7, T47-D, and ZR-75-1), AR positive (MDA-MB-453) and ERα-negative (SKBR3, SUM159, MDA-MB-435, MDA-MB-435, MDA-MB-231, and LM-2) cells. **(D)** The heatmap represents the ChIP-seq tag density for the FOXA1, GATA3, NR2F2, H3K4me1, and H3K27ac DNase-seq results obtained for the MCF-7 cells, ChIP-seq for H3K27ac and H3K4me1, and ATAC-seq for MDA-MB-231 cells at the defined groups characterized by the Venn diagram. **(E)** The average density distribution of H3K4me1 and H3K27ac among the seven different combinations of the three transcription factors in the MDA-MB-231 cell line. **(F)** Kaplan-Meier analysis of metastasis based on the GATA3, NR2F2, and FOXA1 expression levels in the patients. High transcript levels of the three transcription factors were correlated with improved lung metastasis-free survival (LMFS) in breast cancer patients. Analysis of eight groups established by the combination of the three expression levels showing that high expression of all three factors can synergistically improve LMFS. The TF order is FOXA1, NR2F2, and GATA3. For example, E110 indicates patients with high FOXA1 and NR2F2 expression relative to the average value and low GATA3 expression.

Based on the importance of the three factors for ER binding, we assessed the necessity of their expression and binding for ER between ER positive and ER negative breast cancer. First, to confirm that the factors collaborated with the ER, we performed a *de novo* motif analysis using the Homer software. This analysis revealed that 52%, 8% and 29% of the ER cistrome contain the DNA motifs of FOXA1, the GATA family and NR2F2, respectively, with very significant p-values (Figure 5A).

Analysis of the expression profiles of 404 breast cancer patients by integrating three available microarray datasets (GSE2034, GSE2603, GSE5327) obviously showed that these TFs were significantly co-expressed with ERα (co-expression coefficient=0.88) and other hormone receptors, such as the PR and AR, through unsupervised clustering analysis (Figure 5B). Clusters of these factors mostly divided the patients into the ER positive and ER negative subtypes. This result was further supported by western blotting analysis in MCF-7, T47-D, ZR-75-1, MDA-MB-453, SKBR3, SUM-159, MDA-MB-435, MDA-MB-231 and LM2 breast cancer cells, which demonstrated co-expression of ER and these three factors at the protein level (Figure 5C). Interestingly, MDA-MB-453, which is an ER negative, AR positive cell line, also showed a high expression trend of the TFs. According to the above results, the expression levels of the three TFs in TNBC were very low. Thus, we used the binding sites of the seven groups as potential binding sites in MDA-MB-231 cells (a typical cell model of triple-negative breast cancer) to explore the possible chromatin status and epigenetic modifications at these regions in MDA-MB-231. Compared with those of the MCF-7 cells, the histone modifications and DNA accessibility of these sites at MDA-MB-231 had a relatively low signal and showed few changes and non cooperativity among the different combinations (Figure 5D and Figure 5E), which potentially illustrated that regions important for ERα function in MCF-7 cells were condensed in MDA-MB-231 cells.

### NR2F2, FOXA1 and GATA3 associate with low metastases in ERα positive patients

The ERα drives proliferation in more than 70% of all breast cancers. Accordingly, it serves both as a therapeutic target and a prognostic factor. ER positive patients show relatively low invasion and metastases. However, tamoxifen appears to reduce the risk of ER positive breast tumours but to increase the risk of ER negative patients 60. Loss of function of ERa during endocrine treatment for ER positive breast is associated with ER negative metastatic relapse. Recent study show that ERα has been shown to inhibit breast cancer metastasis *in vivo* and *in vitro* 61. Because of the high correlation between the ER and the three genes in ER positive patients and the almost complete lack of their expression in ER negative patients, we speculate that these three genes contributed significantly to the low metastases of ER positive patients. Thus, we assessed the prognostic value of the three genes alone. We performed a survival analysis using microarrays of 404 breast tumours with lung metastasis follow-up. We classified the patients according to expression relative to the mean expression value. High transcript levels of NR2F2, GATA3, and FOXA1 were correlated with improved metastasis-free survival (Figure 5F). The patients were divided into eight groups based on whether the expression levels of the three genes were higher than the corresponding mean expression value. Because the numbers of patients with only high GATA3 and NR2F2 expression (E011) and only high GATA3 (E001) expression were too small, we removed these two groups. Taken together, our results show that NR2F2, GATA3, and FOXA1 can not only be used as markers for metastasis alone but are synergistic in inhibiting breast cancer metastasis, thereby contributing to the relatively low level of metastasis in ERα positive breast cancer compared with negative breast cancers.

### The relative hypermethylation of promoter regions of the three genes in ERα negative patients contributes to the low expression

**Figure 6:**
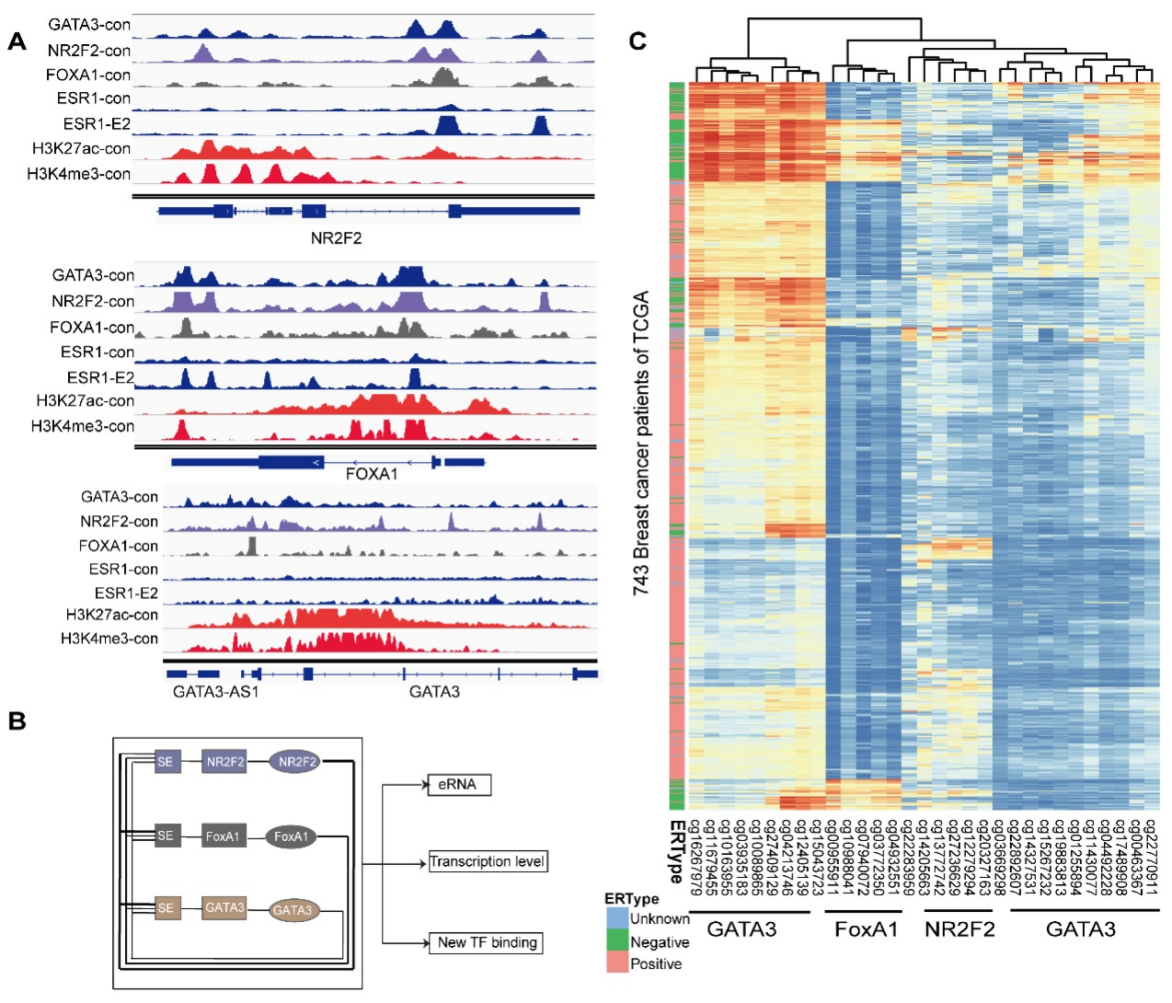
** The relative hypermethylation of promoter regions at the three genes in ER negative patients contributes to the low expression. (A)** The IGV shows the ChIP-seq signals of H3K4me3 and H3K27ac and the corresponding transcription factors at the NR2F2, FOXA1, GATA3 and ERα gene loci in the MCF-7 cell line. **(B)** Schematic diagram showing that genes encoding these three factors are themselves driven by super-enhancers and form an interconnected feedback loop. **(C)** Unsupervised clustering of 743 breast cancer patients from TCGA with the methylation probes assigned to the three genes (NR2F2, FOXA1 and GATA3), showing that methylation of the promoter regions of the genes is inversely related to the ERα histological status. Each row represents a patient, and each column represents a probe. The relative level of DNA methylation (normalized and transformed beta value) is represented with the colour bar.

The concurrent expression of GATA3, NR2F2, and FOXA1 in ERα positive breast carcinomas is intriguing. Nevertheless, this finding fits well with our working model that GATA3, NR2F2 and FOXA1 are functionally necessary for ER positive patients. The question is how GATA3, NR2F2 and FOXA1 expression is all downregulated or concurrently lost in ER negative breast cancer. To further examine the underlying reason, we inspected the binding profiles of transcription factors that might play a potential role using IGV tools. We found that all three factors bound as a group to the promoter of each one (Figure 6A) and that these three TFs formed an interconnected autoregulatory loop (Figure 6B). This approach may represent a smart strategy to ensure the balanced co-regulation of gene networks for precise regulation of important transcription programs that are extremely sensitive to biological processes.

In the mammalian genome, DNA methylation is an epigenetic mechanism involving the transfer of a methyl group onto the C5 position of the cytosine to form 5-methylcytosine. DNA methylation regulates gene expression by recruiting proteins involved in gene repression or by inhibiting binding of transcription factor(s) to DNA. We collected methylation 450K microarrays for more than one thousand breast cancer patients from TCGA (The Cancer Genome Atlas). After filtering the data, we retained 773 effective patients with more than 40 methylation sites across the promoters of the three genes. Unsupervised free clustering showed that the CpG sites in the promoters of these three genes tended to have relatively low methylation levels in ER positive breast cancer and hypermethylation in ER negative patients. The hypermethylation in promoter regions suppressed binding of the transcription factors, including POLII, and thus inactivated transcription of the three TFs (Figure 6C). Our results may well illustrate the co-expression of NR2F2, GATA3, and FOXA1 in ER positive breast patients and concurrent low expression in ER negative ones.

## Discussion

ER interact with numerous associated proteins that can help to tether the receptor to DNA including pioneer factor and that can keep optimal open DNA and that can possess intrinsic enzymatic activity. Here, by screening the interactome of the ER and other hormone receptors to explore candidate interactions, we found NR2F2 is required for growth and binds to most sites independently of estrogen. NR2F2 is required for ER binding and inhibition of this protein decreases ATAC-seq signal. NR2F2 acts as a functional co-factor of ERα cobinding with pioneer factor FOXA1 and GATA3 by binding ER-related enhancers. In addition, we demonstrated that sites occupied by all three TFs were associated with the highest chromatin opening and ER recruitment degrees. NR2F2, GATA3, and FOXA1 jointly cover 85% of ER binding sites.

**Figure 7:**
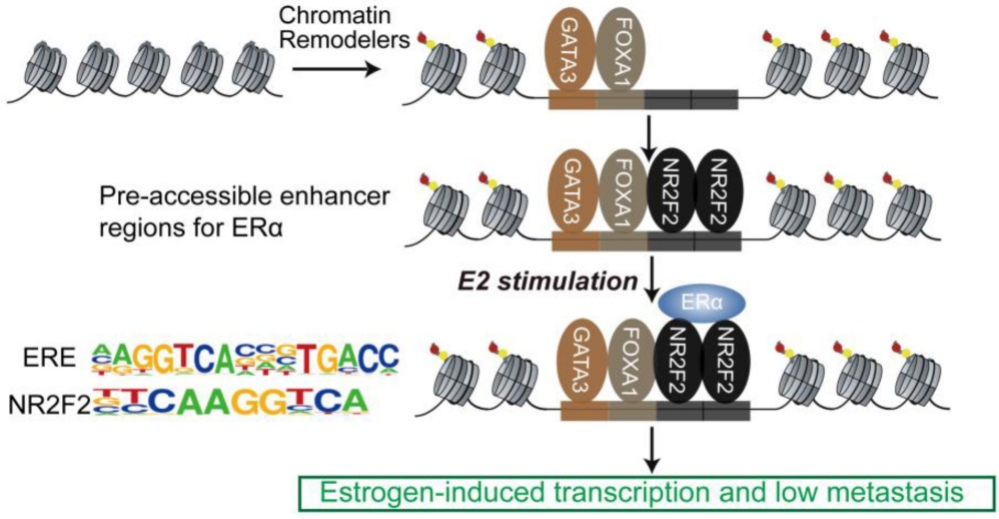
**Proposed mode of utilization of preformed open enhancer chromatin by ER during effect of estrogen.** The majority of ERα binding sites are found within super-enhancers harboring ER motifs that are co-occupied by GATA3, NR2F2, and FOXA1 before the ERα is recruited to the chromatin.

The sequential binding of NR2F2, GATA3, and FOXA1 in ER binding events is still undetermined. Previously, it was reported that GATA3 acts upstream of FOXA1 when mediating ESR1 by shaping the enhancer accessibility 16. Depletion of GATA3 reduce partial ER binding by reducing specific FOXA1 binding and histone modification deposition 16. Our data showed that there was no significant change in the binding of FOXA1 after knocking out NR2F2, potentially indicating that the binding of NR2F2 had little effect on FOXA1. More work and research is still needed to further demenstrate FOXA1 act upstream of NR2F2 (Figure 3I and Figure 7). And ER and NR2F2 share a part of the motif, and there is a certain similarity between their motifs. Based on the existing results and reports, we speculate the model may like this (Figure 7): pioneer factors GATA3 and FOXA1 first bind on nucleosome chromatin, establishing open chromain and then NR2F2 bind to help maitain accessible chromatin. This prepare pre-existing open enhancer landscape for ER. We propose that upon E2 stimulation, the ERα binds to the pre-existing enhancer landscape co-bound by cofactor NR2F2 and pioneer factors GATA3, NR2F2 instead of *de novo* established accessible chromatin to drive the complement of the ERα core function at short time.

The expression of these three transcription factors can mostly define the positive or negative ER status. Low levels of these three TFs were strongly associated with an ER negative status, PR-negative status, poor survival, positive lymph node status, higher histological grade, and increased risk for recurrence and metastasis. They also had high expression levels in model ER-AR+ cell line MDA-MB-453 cells. Our data shown that AR can function as prognostic targets in ER negative patients but has no effect on ER positive ones (Figure S2B and S2C). New treatments that resistant receptor activity and pioneer factor function can be combinatorially suppressed are recommended for endocrine therapy resistant patients. The new subtype of breast cancer, termed Molecular Apocrine, has been characterized. Molecular apocrine tumors are ER negative, but AR positive and in many cases they express genes that are normally expressed in ER-positive luminal tumors, including XBP-1 and FOXA1 62, 63. Our investigation may provide valuable strategies for ER resistant patients and ER negative even triple negative but AR positive therapeutic targets.

Among these three factors, FOXA1 and GATA3 are important transcription regulatory proteins and known ER-interacting transcription factors. GATA3 is reported to be highly expressed in the mammary epithelium and is expressed exclusively in the luminal epithelial cell population, where it specifies and maintains luminal cell differentiation. Inactivation of GATA3 results in failure of the mesenchymal-epithelial transition (MET), which increases breast cancer metastasis.

Epigenetic silencing of tumour suppressor genes by DNA methylation is a hallmark of tumours 64. Strong evidence exists for a subgroup of tumours that exhibit concordant tumour-specific DNA methylation: the ''CpG island methylator phenotype” (CIMP). The CIMP has been reported to be useful for prognostic predictions and is associated with a worse patient prognosis and response to treatment in a variety of tumour types, including bladder, breast, endometrial, gastric, glioblastoma, hepatocellular, lung, ovarian, pancreatic, renal cell and leukaemia. Comprehensive molecular characterization and the molecular causes underlying the CIMP have been relatively well studied for glioblastoma and renal cell carcinoma. However, evidence remains insufficient to interpret the function of the CIMP in human breast cancer, and new clinical CIMP markers for diagnosis and prognosis are still required. In our analysis, we found that hypermethylation in the GATA3, NR2F2, and FOXA1 promoters was inversely correlated with the ER positive histological state. Differentially methylated regions within the three genes resulted in differential expression among the investigated subgroups and were correlated with metastasis-free survival. Our findings may provide implications for the treatment of ER negative patients. DNA-demethylating agents activate tumour suppressor genes that are silenced by DNA methylation in cancer. 5-Aza-2′-deoxycytidine (5-azaCdR), which is a deoxy-analogue of 5-azacytidine, can induce methylated pro-metastatic genes by DNA demethylation and induce cancer cell invasiveness. Consistent with our research, 5-aza-2-deoxycytidine has been reported to reduce NR2F2 methylation and increase NR2F2 expression to restore anti-oestrogen sensitivity in resistant breast cells 65. Thus, we may use an inhibitor of DNA methylation to increase hypermethylation of the three genes and reduce metastasis of ER negative cancers.

Taken together, our results show that the interplay of cofactor NR2F2 with pioneer factors GATA3 and FOXA1, which promote an open chromatin structure, subsequent nuclear receptor binding, and resultant initiation of E2-induced transcriptional programmes, are essential for optimal ER binding to the DNA. We further uncovered the functional importance of an enhanceosome that exerted significant combinatorial control over the transcriptional network related to proliferation and metastasis of ER positive breast cancer cells.

## Supplementary Material

Supplementary figures and supplementary table 1.

Supplementary table 2.

Supplementary table 3.

Supplementary table 4.

Supplementary table 5.

## Abbreviations

PF: Pioneer factor
ERE: Estrogen responsive element
ERα: Estrogen receptor α
ENCODE: The Encyclopedia of DNA Elements
ChIP-seq: Chromatin immunoprecipitation followed by sequencing
ChIP-qPCR: Chromatin immunoprecipitation followed by quantitative real-time PCR
ATAC-seq: Assay for Transposase Accessible Chromatin with high-throughput sequencing
TCGA: The Cancer Genome Atlas
GRO-seq: Global run-on sequencing
