# Supplementary material for: Cooperativity of co-factor NR2F2 with Pioneer Factors GATA3, FOXA1 in promoting ERα function
Source: Theranostics. 2019 Aug 21;9(22):6501–16. doi: 10.7150/thno.34874 (PMC6771234; doi:10.7150/thno.34874)
Supplement: Supplementary file 1 — Supplementary figures and supplementary table 1. [file thnov09p6501s1.pdf]

Figure S1

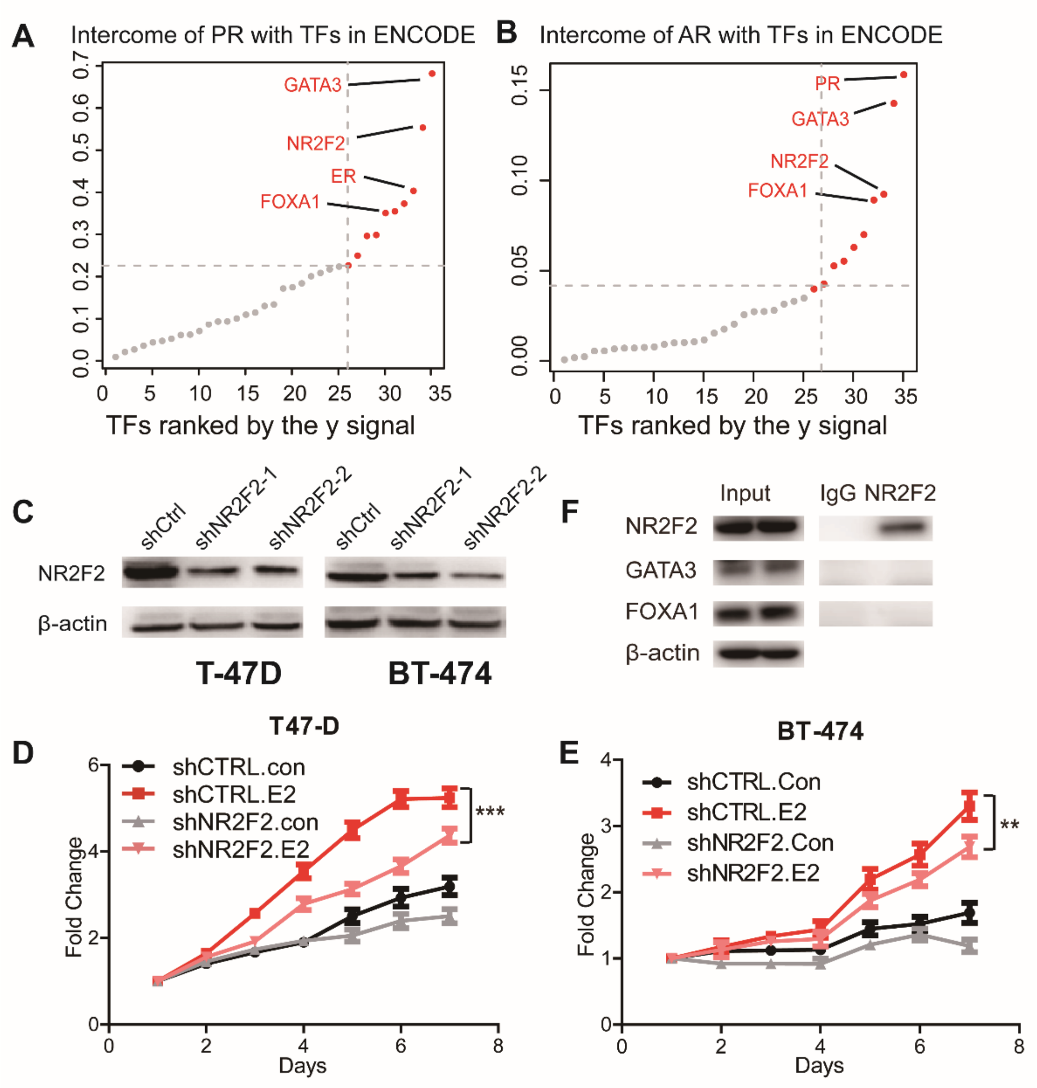

(A, B) Distribution of the overlap (value of y) of all transcription factors with the AR and PR.

(C) NR2F2 stable depletion via lentivirus-delivered shRNA reduces its protein levels in both BT-474 and T47-D cell lines.

(D, E) T-47D and BT-474 breast cancer cells depleted of NR2F2 fail to proliferate in response to oestrogen stimulation compared to cells subjected to the control treatment to a certain degree.

Figure S2

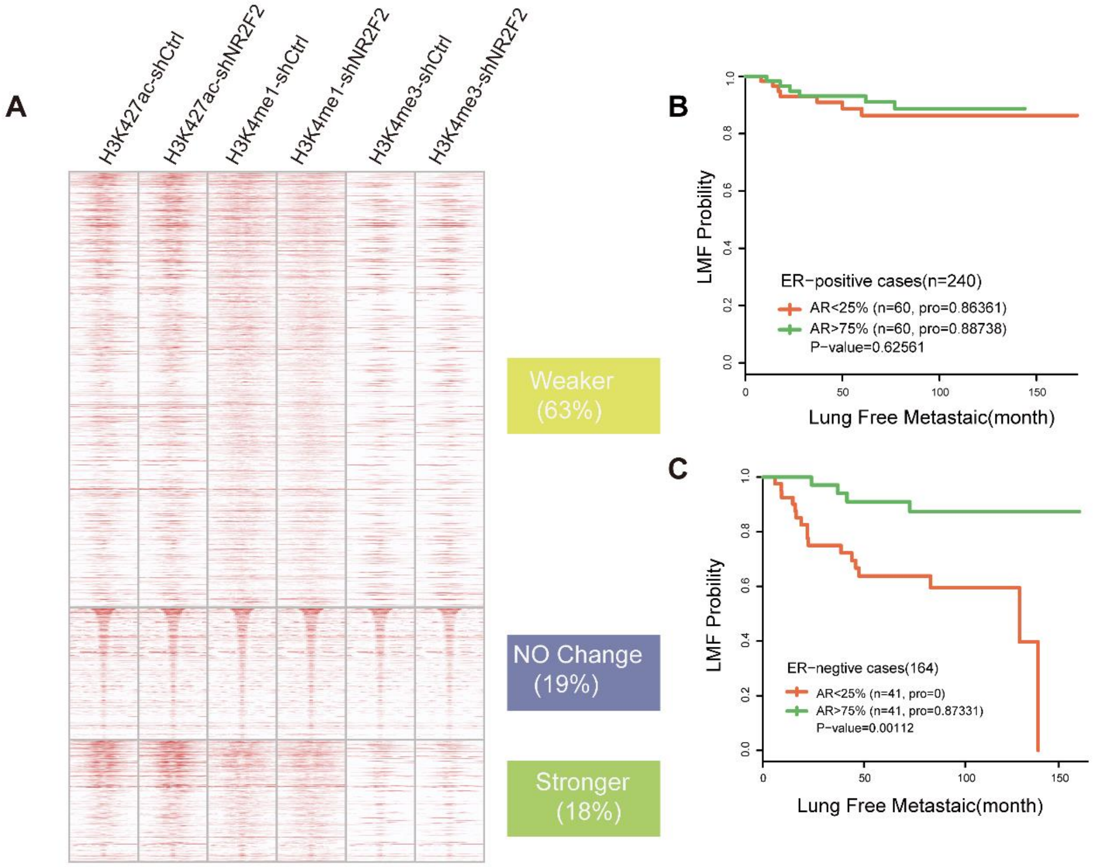

(A) Heatmap analyses of ChIP-seq signals of histone modification (H3K27ac, H3K4me3 and H3K4me1)in the control and shNR2F2 MCF-7 cells, ranked by ChIP-seq signals of ER. All ChIP-seq signals are displayed from -2.5 kb to +2.5 kb surrounding the center of each annotated ER peak.

(B, C) Kaplan-Meier product limit analysis of lung metastasis probility in ERa-positive and ERa-negative breast cancer patients, respectively. Patients were dichotomized by the median percent AR-positive nuclear area (>75% and <25%)

Supplementary Table 1. Data Used in analysis.

ChIP-Seq	MCF7			MDA-MB-231
	FOXA1.E2	HDAC2	RAD21	H3K4me1
	FOXA1.Con	MYC	ESRRA	H3K27ac
	GATA3.E2	TCF12	CTCF	
	GATA3.Con	SIN3A	MTA1	
	ER.E2	TCF12	SRF	
ChIP-Seq	ER.Con	MAX	MAZ	
	H3K4me1.E2	JUND	EGR1	
	H3K4me1.Con	FOXM1	PML	
	H3K4me2.E2	E2F1	REST	
	H3K4me2.Con	FOSL2	GABPA	
	H3K27ac.E2	TEAD4	RFX5	
	H3K27ac.Con	MNT	MAFK	
	CEBPB	TCF7L2	GTF2F1	
	NR2F2	ELF1	HSF1	
	PR	ZNF217	TAF1	
	CEBPb			
	AR			
	NR2F2-Con	H3K4me3-shCtrl		
	NR2F2-E2	H3K4me3-shNR2F2		
	ER-shCtrl-E2	H3K4me1-shCtrl		
	ER-shNR2F2-E2	H3K4me1-shNR2F2		
RNA-Seq	shNR2F2-con			
	shCtrl-con			
	shNR2F2-E2.12h			
	shCtrl-E2.12h			
ATAC-Seq	ATAC-Seq-shCtrl			ATAC-seq
	ATAC-Seq-shNR2F2			
GRO-Seq	GRO-Seq.E2			
	GRO-Seq.Con			
DNase-Seq	DNase-Seq.E2			
	DNase-Seq.Con			

Note:data used in black font is downloaded from public dataset and in blue is newly generated by our lab.
